# Effectiveness of Music Therapy with Personalized Rhythmic Auditory Stimulation Plus Music-Contingent Gait Training in Patients with Parkinson’s Disease: A Systematic Review

**DOI:** 10.3390/neurolint18020026

**Published:** 2026-02-03

**Authors:** Andrea Demeco, Rosa Cristina Bruno, Raffaele Bonfiglio, Lorenzo Mancini, Federica Pisani, Lorenzo Scozzafava, Chiara Conte, Antonio Ammendolia, Alessandro de Sire, Nicola Marotta

**Affiliations:** 1 Physical and Rehabilitative Medicine, Department of Medical and Surgical Sciences, University of Catanzaro “Magna Graecia”, 88100 Catanzaro, Italy; andrea.demeco@unicz.it (A.D.); rosacristina.bruno1@studenti.unicz.it (R.C.B.); raffaele.bonfiglio@studenti.unicz.it (R.B.); lorenzo.mancini@studenti.unicz.it (L.M.); f.pisani@unicz.it (F.P.); lorenzo.scozzafava@studenti.unicz.it (L.S.); chiara.conte@studenti.unicz.it (C.C.); ammendolia@unicz.it (A.A.); 2 Research Center on Musculoskeletal Health, MusculoSkeletalHealth@UMG, University of Catanzaro “Magna Graecia”, 88100 Catanzaro, Italy; nicola.marotta@unicz.it; 3 Physical and Rehabilitative Medicine, Department of Experimental and Clinical Medicine, University of Catanzaro “Magna Graecia”, 88100 Catanzaro, Italy

**Keywords:** RAS, Parkinson, auditory cueing, music-based, music therapy, freezing of gait, treadmill

## Abstract

**Background**: Parkinson’s disease (PD) is characterized by motor disturbances that significantly impact balance, gait, and quality of life. Personalized Rhythmic Auditory Stimulation (pRAS) is an emerging rehabilitative approach that utilizes auditory entrainment to improve step and gait control. The aim of this systematic review is to critically summarize the data from the most recent evidence concerning the use of pRAS in gait rehabilitation for patients with Parkinson’s disease. **Methods**: A systematic review was conducted following PRISMA guidelines, including records that evaluated music-based or technological interventions based on personalized RAS. Primary outcomes included spatiotemporal gait parameters and distance covered. **Results**: Ten studies were included in the analysis. All the studies reported clinically relevant improvements: increases in gait speed, step length, and amplitude. Moreover, a reduction in freezing of gait episodes (up to 36%), greater walking distance, and good adherence were reported. **Conclusions**: Personalized, adaptive, or on-demand solutions proved more effective than traditional forms of cueing. Moreover, the available evidence suggests that pRAS constitutes an effective and safe rehabilitative option for gait disturbances in PD. However, further studies with larger sample sizes and prolonged follow-up periods are necessary to evaluate its long-term impact and transferability into clinical practice.

## 1. Introduction

Parkinson’s disease (PD) is a prevalent, progressive neurodegenerative disorder primarily defined by its cardinal motor symptoms: rest tremor, bradykinesia, rigidity, and postural instability [1,2]. The underlying pathophysiology involves a dysfunction within the basal ganglia-thalamocortical loops, impairing the internal generation of motor timing and sequencing necessary for sustained, rhythmic ambulation [3]. Among the most debilitating manifestations, the gait disturbances are the most common, with symptoms including decreased gait speed, shortened stride length, increased step variability, and the intermittent yet highly disruptive episodes of Freezing of Gait (FOG) [4]. These locomotor impairments severely compromise patient autonomy, elevate the risk of falls, and significantly diminish the overall quality of life [5]. Traditional pharmacological treatment and deep brain stimulation have shown interesting results in the treatment of motor symptoms, but they provide limited improvement in gait dysfunctions, particularly FOG [6]. In this context, rehabilitation could play a role in synergy with the other therapies to restore effective walking patterns and reduce the risk of falls in PD patients [7].

In particular, rehabilitative interventions providing external sensory cues, e.g., specifically rhythm and music, have gathered significant research interest. Rhythmic Auditory Stimulation (RAS) is a technique employing regular acoustic signals (such as a metronome beat or rhythmic music) to provide an external temporal timing to follow. While standard RAS relies on metronome-like pulses to provide a stable temporal cue for gait, personalized RAS (pRAS) represents an advancement by dynamically adjusting auditory cues to the patient’s specific cadence, preferences, and individual gait phase [8].

Auditory-motor entrainment, or audio spinal coupling, is the innate ability to synchronize rhythmic body movements with external auditory rhythms, like a musical beat [9]. Numerous studies and previous systematic reviews have consistently demonstrated that standard RAS can improve key spatiotemporal gait parameters, including enhancing walking velocity, increasing stride length, and reducing step-to-step variability, thus promoting a more stable and less energy-demanding gait [10,11].

However, an evolution of RAS is the development of personalized RAS (pRAS), often integrated and enhanced with Music-Contingent Gait Training (MCGT); although MCGT refers to protocols where music serves as the primary driver of the gait training, it is fundamentally situated within the broader framework of Neurologic Music Therapy (NMT), a comprehensive, evidence-based system—encompassing techniques such as RAS—delivered by certified therapists [12,13].

Unlike fixed-rate metronomes, pRAS systems are tools designed to overcome the limitations of conventional cueing by calibrating the time, structure, and musical complexity to the unique motor phenotype and functional reserve of the individual patient [14]. This involves measuring a patient’s self-selected cadence (preferred walking rhythm) and then setting the auditory cues slightly above this rate (typically 5%−25% faster) to maximize the rehabilitation outcome [15].

Furthermore, advanced pRAS and MCGT systems incorporate adaptive or on-demand cueing, dynamically adjusting the rhythm based on real-time feedback (e.g., from inertial sensors) to address specific moments of motor difficulty, such as the onset of FOG episodes [16]. This level of customization is hypothesized to amplify the strength and duration of audio-motor synchronization, potentially leading to superior and more sustained rehabilitative effects compared to generic or non-adaptive protocols [16,17]. Given the complexity of PD gait and the promise of targeted rehabilitation, a comprehensive synthesis of the latest high-quality evidence is crucial to establish the effectiveness of these tailored interventions [14,17].

Therefore, this systematic review aimed to assess the effectiveness of personalized pRAS with MCGT in improving spatiotemporal gait parameters in patients with Parkinson’s disease.

## 2. Materials and Methods

### 2.1. Data Sources and Searches

PubMed, Scopus, Web of Science, and Google Scholar databases were systematically searched for English-language articles published from the inception until 20 November 2025 according to each specific thesaurus, following this string strategy: (“Parkinson disease”) OR (Parkinson) OR (PD) AND ((“rhythmic auditory cueing”) OR (“rhythmic auditory stim*”) OR (RAS) OR (“music rehabilitation”) OR (rhythm*) OR (rhythmic) OR (“music therapy”) OR (melody) OR (beat) OR (metronome) OR (“rhythmic auditory stimuli”) OR (“music therap*”) OR (music) OR (tone) OR (music therapy [MeSH Terms])) AND ((capture*) OR (wear*) AND ((movement) OR (motion) OR (motor*)). This systematic review was conducted according to the guidance of Preferred Reporting Items for Systematic reviews and Meta-Analyses (PRISMA) [18]; the associated checklist is available in the Supplementary Materials (File S1). The following systematic review was registered on the PROSPERO registry with the code CRD420251129636.

### 2.2. Study Selection

After importing all search entries into a new Zotero (VA, USA) library, the exclusion process began with two phases. First, duplicate records were automatically removed using Zotero’s built-in function; the remaining articles underwent a screening process to assess their eligibility for inclusion in this systematic review. This phase was conducted independently by two reviewers (RF and LM), who each created a separate list of included and excluded articles. These lists were then exported as a CSV file for analysis in Microsoft Excel. In cases of disagreement between the two reviewers on an article’s eligibility, a third reviewer (RCB) was consulted to reach a final consensus.

Studies were considered eligible if responding to the questions defined by the following PICO model:

(P) Participants: Patients with Parkinson’s disease;

(I) Intervention: pRAS with MCGT;

(C) Comparator: Conventional therapy;

(O) Outcome measure: Gait parameters.

(S) Study design: experimental, quasi-experimental, and observational study designs (e.g., cross-sectional, longitudinal, and case-control studies). The inclusion of both randomized controlled trials and observational studies was intended to provide a comprehensive overview of the effects of pRAS, capturing evidence derived from both controlled experimental conditions and real-world clinical settings, where randomized designs might be limited or not feasible. This inclusive approach allowed for a comprehensive understanding of the effects of core stability training, regardless of the level of control or the type of analysis. Specifically, pRAS was applied using rhythmic cueing calibrated to the patient’s self-selected cadence and a sensor-based dynamic system that adapts rhythmic cues in real time.

To further refine our selection and maintain the focus and quality of the review, only studies with full-text availability were included to allow for a complete and detailed assessment of methodologies and results, preventing reliance on potentially incomplete information.

### 2.3. Data Extraction

Data from the selected studies were independently extracted by two reviewers (RF and LM) using a custom template. Any disagreements were resolved by consulting a third reviewer (RCB) to reach a consensus. Key information about the studies was summarized in tables (created with Microsoft Word 2021). The following data were extracted: (1) first author; (2) publication year; (3) nationality; (4) age of study participants; (5) population and the number of patients included; (6) main limitations; and (7) main findings.

### 2.4. Data Synthesis and Quality Assessment

Two independent reviewers (RF and LM) performed the risk of bias assessment of the included studies. The JBI—the Joanna Briggs Institute tool—was used to assess the methodological quality of case control studies, cohort studies, cross-sectional studies, and case series [19]. Each study was assessed according to predefined criteria. The JBI model of evidence-based healthcare conceptualizes evidence-based practice as clinical decision-making that considers the best available evidence; the context in which care is delivered; client preference; and the professional judgement of the health professional [20]. Any disagreements regarding its evaluation were resolved with the involvement of a third reviewer (RBC).

## 3. Results

### 3.1. Study Characteristics

**Figure 1:**
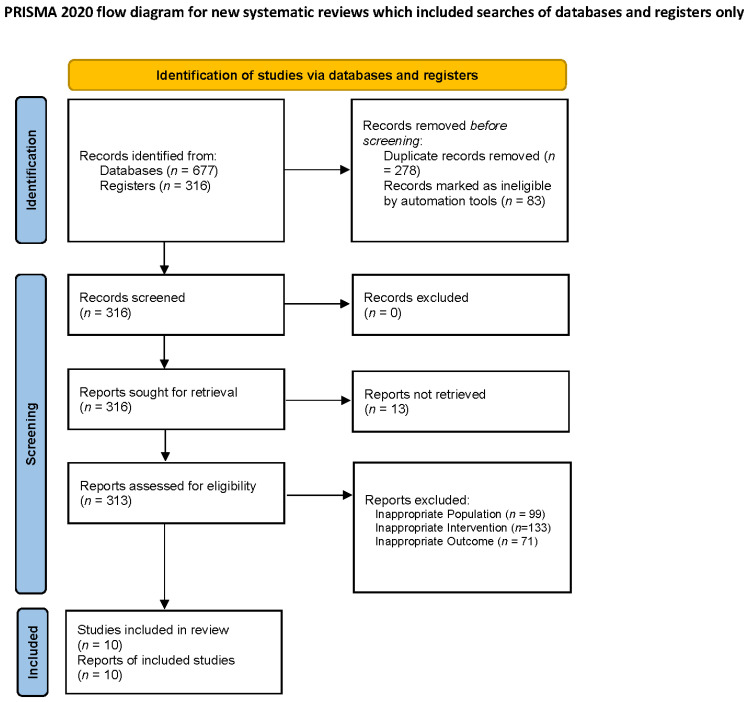
PRISMA flow chart.

From the initial 677 studies, after the removal of duplicates, we analyzed the full text of 21 records, as depicted in Figure 1.

Finally, 10 articles were included in our review [21,22,23,24,25,26,27,28,29,30] (see Table 1 for further details).

### 3.2. Characteristics of the Studies’ Interventions

This systematic review included studies originating from several countries (Canada [21,22], Thailand [23], Poland [24], Italy [25,26,27], USA [28,30], Romania [29]), suggesting a global interest in personalized music therapy for PD rehabilitation. Despite the geographical spread, the study groups exhibit notable homogeneity concerning disease severity. The number of patients included ranged from 21 [28] to 55 [24].

We included six randomized controlled trials (RCT) [22,23,25,26,27,30] three pilot studies [21,24,29], and one prospective crossover study [28].

Studies focus on patients with mild to moderate PD (Hoehn & Yahr scale, HY II–III), indicating that musical intervention is primarily studied in early phases with patients still responsive to external cues.

In this scenario, de Bruin et al. [22] and Calabrò et al. [26] utilized music as a cadence-matched metronome or individualized rhythmic cues (RAS) on a treadmill or cycle, typically progressing up to 120 beats per minute (bpm). The goal was to leverage the rhythmic entrainment property to improve the spatio-temporal parameters of gait. De Luca et al. [27] combined RAS (music + bell cues) with training on a robotic treadmill (Biodex Gait Trainer 3), highlighting the integration of motor technology with rhythmic stimulation. Chawla et al. [28], in a single-session, crossover study, explored the effect of different rhythmic frequencies (85%, 100%, 115% of spontaneous cadence) and participant-selected music, underscoring the importance of personalizing the cue. Bukowska et al. (2016) [24] employed a Neurologic Music Therapy (NMT) program, which included the association of TIMP (Therapeutic Instrumental Music Performance) and PSE (Patterned Sensory Enhancement), as well as pRAS applied not only to gait but also to activities of daily living (ADLs), balance, and stability. Lastly, Spina et al. (2016) [25] and Fodor et al. (2021) [29] adopted an approach of active music therapy, focused on music production, singing, and dancing, for 24 weeks, with an emphasis on holistic and social benefits, in addition to motor ones.

Regarding study duration and design, Chaiwanichsiri et al. [23] and Bukowska et al. [24] utilized intensive programs of 4 weeks. Most studies ranged between 6 and 13 weeks (Burt et al. [21], Calabrò et al. [26], De Luca et al. [27], Porciuncula et al. [30]). Nevertheless, Spina et al. [25] stands out with an extended 24-week program of active music therapy. Most studies employed RCTs or, as in Porciuncula et al. [30], compared an experimental group (Amped-PD, using adaptive music-based RAS) with an active control group (brisk walking without cues) to isolate the specific efficacy of the musical intervention.

In summary, the comparative analysis highlighted a strong consensus on the use of rhythmic stimuli to improve PD gait. While RAS remained the foundation, more recent studies, particularly those from Italy and North America, are moving toward more sophisticated interventions such as Music-Contingent Gait Training and NMT. The trend is to personalize the rhythmic cue and integrate it with specific physical training (often on a treadmill), offering a promising framework for future evidence-based rehabilitation guidelines.

### 3.3. Outcome

The data focused on assessment of motor function and, specifically, objective analysis of gait; measures such as gait velocity/speed, stride/step length, and cadence were widely utilized by de Bruin et al. [22], Chaiwanichsiri et al. [23], Bukowska et al. [24], Chawla et al. [28], and Porciuncula et al. [30] to quantify intervention-induced changes in locomotion.

For the evaluation of functional mobility and dynamic balance, the Timed Up and Go Test (TUG) emerged as the most widely shared outcome, having been employed by Chaiwanichsiri et al. [23], Spina et al. [25], Calabrò et al. [26], De Luca et al. [27], and Porciuncula et al. [30]. Other specific functional tests were utilized to complement the TUG: the 10-Meter Walk Test (10MWT) and the 6-Minute Walk Distance (6MWD) were included to measure speed or endurance in the studies by Chaiwanichsiri et al. [23], Calabrò et al. [26], De Luca et al. [27], and Porciuncula et al. [30], while scales such as the Functional Gait Assessment (FGA) and the Berg Balance Scale (BBS) allowed for a more detailed evaluation of balance and gait disability [26].

Regarding the overall impact on the disease, almost all studies utilized the motor section of the Unified Parkinson’s Disease Rating Scale (UPDRS-III) or its revision, the MDS-UPDRS, as the clinical gold standard measure of motor symptom severity in PD [21,22,25,26,30]. Finally, to assess the patient’s perceived impact and quality of life (QoL), several studies utilized the Parkinson’s Disease Questionnaire (PDQ-39) or its related domains [25,29,30].

Gait speed was among the most frequently assessed parameters and generally showed improvements following pRAS-based interventions, particularly in studies employing individualized or adaptive rhythmic cues combined with treadmill or technology-assisted gait training [23,26,27,30]. Step length and stride length were also commonly evaluated and tended to improve in response to rhythmic cueing, with more pronounced effects observed when auditory cues were calibrated to the patient’s spontaneous cadence or dynamically adjusted in real time [21,24]. Cadence-related effects varied depending on cueing strategy: cueing set above spontaneous walking frequency was associated with increased cadence, whereas cadence-matched or adaptive cueing primarily promoted gait regularity and stability rather than speed [28]. Studies adopting music-contingent or active music therapy approaches reported more variable motor outcomes, with some interventions emphasizing feasibility, adherence, or psychosocial benefits rather than robust changes in spatiotemporal gait parameters [22,25,29]. Although methodological heterogeneity and variability in outcome measures precluded quantitative pooling of results, the overall direction of effects across studies supports a beneficial role of personalized rhythmic cueing on gait-related outcomes in patients with mild to moderate Parkinson’s disease [21,23,24,26,27,30].

#### 3.3.1. Spatiotemporal Gait Parameters and Endurance

Regarding cadence and step/stride length, RAS consistently demonstrated a significant increase in cadence (steps/minute) and stride length. De Bruin et al. [22] reported an average increase in cadence of 3.8% and stride length of +10.7%. Chaiwanichsiri et al. [23] showed comparable improvements in cadence (+3.1%) and a notable increase in stride length (12%). Bukowska et al. [24] also found significant improvements in both cadence (*p* = 0.019) and stride length (*p* = 0.015). Chawla et al. [28] confirmed the high controllability of cadence by successfully manipulating participants’ rhythm to align with auditory cues at various frequencies (85%, 100%, and 115% of baseline cadence). Regarding gait speed, the increases in velocity and endurance parameters were direct functional outcomes. De Bruin et al. [22] documented an increase in walking speed of +11.3%. Chaiwanichsiri et al. [23] reported that the music + treadmill group achieved the largest increase in walking speed (8.6%). Bukowska et al. [24] reported a significant improvement in walking speed (*p* = 0.005) following NMT. Functional endurance, measured by the 6-Minute Walk Distance (6MWD), showed significant benefits: Porciuncula et al. [30] found that the RAS group demonstrated an increase of +16.9 m in the 6MWT compared to the control group, specifically under dual-task conditions.

#### 3.3.2. Comparison of Timed Up and Go (TUG) Test Results

The Timed Up and Go (TUG) test, a functional measure of mobility, balance, and transitions, yielded conflicting results across the studies analyzed. Chaiwanichsiri et al. [23], Calabrò et al. [26], and De Luca et al. [27] reported a significant improvement in TUG scores (*p* < 0.05) after rhythmic auditory interventions, suggesting a positive transfer of rhythm to dynamic balance tasks and functional mobility. Conversely, Spina et al. [25] explicitly reported no significant beneficial effects on TUG scores immediately post-intervention, further noting a significant deterioration at the 3-month follow-up. This overall responsiveness of the TUG, despite the exception in Spina et al. [25], highlighted that RAS could effectively optimize not only the rhythmic and spatial aspects of walking but also its translation to the complex functional components of mobility, like turning and transitions, which are fundamental for reducing fall risk [26,27].

#### 3.3.3. Motor Severity (UPDRS-III) and Quality of Life (QoL)/Disability

##### Unified Parkinson’s Disease Rating Scale (UPDRS-III)

Clinical assessment of overall motor symptom severity, measured using the UPDRS-III, showed statistically significant benefits. Calabrò et al. [26] demonstrated a statistically significant improvement in the UPDRS-III (*p* = 0.001) in the RAS group. This finding is reinforced by Bukowska et al. [24], who also reported a significant improvement in the UPDRS-III (*p* = 0.012) after NMT. The impact on QoL, assessed using the Parkinson’s Disease Questionnaire-39 (PDQ-39), provided heterogeneous and less consistent statistical results [25,29,30]. However, measures of perceived functional stability showed clearer effects.

Moreover, regarding the fear of falling, Calabrò et al. [26] reported a highly significant improvement in the Tinetti Falls Efficacy Scale (TFES) (*p* < 0.001), indicating a robust reduction in the perceived fear of falling. In this scenario, Burt et al. [21] reported a significant improvement in the Freezing of Gait Questionnaire (FOG-Q) score (*p* = 0.01), suggesting a reduction in the severity of perceived freezing episodes.

### 3.4. Certainty of Evidence and Risk of Bias

The certainty assessment, summarized via the GRADE framework, indicates a moderate level of evidence for primary motor outcomes, including spatiotemporal gait parameters, functional mobility, and motor severity. While results for gait speed and cadence were largely consistent—showing increases of up to 11.3% and 3.8%, respectively—the evidence for functional mobility was downgraded due to inconsistency. This was exemplified by the conflicting reports between the significant improvements in TUG scores noted by Calabrò et al. [26] and the lack of effect observed by Spina et al. [25]. Furthermore, quality of life (PDQ-39) outcomes were rated as “very low” due to very serious imprecision and heterogeneous data results across studies. Consequently, while the therapeutic signal for motor improvement remains strong, the clinical impact on subjective well-being requires more robust, large-scale investigation to achieve higher certainty, as depicted in Table 2.

In the present systematic review, we used the JBI Critical Appraisal Checklist for quasi-experimental studies and for case series to assess the risk of bias of the included studies. We reported an overall good methodological quality across the included studies, with total scores ranging from approximately 78% (seven out of nine) to 100% (nine out of nine). Specifically, most studies demonstrated clear group comparability and appropriate statistical analysis. This high level of methodological rigor across the selected literature provides a solid foundation for the subsequent analysis of clinical outcomes.

Most studies—including de Bruin et al. [22], Chaiwanichsiri et al. [23], Spina et al. [25], Calabrò et al. [26], and Porciuncula et al. [30]—achieved the maximum score (100%), indicating very low risk of bias, clear cause–effect sequencing, reliable outcome measurements, and appropriate statistical analyses.

A second group of studies [21,24,27,29] scored approximately 89% (eight out of nine), with the only limitation being incomplete follow-up, while still maintaining an overall low risk of bias. The study with the lowest score was Chawla et al. [28] (approximately 78% or seven out of nine), mainly due to the absence of a control group and some uncertainty regarding treatment comparability across conditions. Overall, studies demonstrated strengths in intervention clarity, reliability of outcome measures, and statistical rigor. The most frequent limitations involved incomplete follow-up and lack of a control group in some protocols. Taken together, methodological quality was high, supporting the robustness of the available evidence while indicating the need for future research with more structured follow-up procedures and stronger controlled designs (for further details, see Table 3).

## 4. Discussion

This systematic review aimed to summarize clinical evidence on the efficacy of RAS, pRAS, and music therapy for improving gait and functional mobility in PD.

Across the reviewed studies, rhythmic-auditory approaches consistently improved spatiotemporal gait parameters, including stride length, cadence, and walking speed, with several trials also reporting benefits in balance confidence, motor severity, and quality of life. The strongest evidence emerged from interventions incorporating personalization, adaptive modulation, or contingent auditory feedback, suggesting that cueing strategies tuned to an individual’s motor output produce more effective entrainment than fixed-tempo RAS [31]. The transition from fixed-tempo Rhythmic Auditory Stimulation (RAS) to personalized and closed-loop technologies (pRAS) represents a paradigm shift in Parkinson’s disease (PD) gait rehabilitation; by utilizing real-time biofeedback and adaptive modulation, these systems synchronize auditory stimuli with an individual’s moment-by-moment motor fluctuations, enhancing ecological validity [32].

Neurophysiological findings from RAS-enhanced training further support these observations, indicating strengthened sensorimotor integration and timing-related neural activity [33].

Interestingly, Spina et al. [25] documented improvements in executive functioning, mood, and quality of life. Moreover, the Ambulosono MCGT platform [21] demonstrated high adherence (97%) and improvements in mood and anxiety in early-stage PD, despite modest motor effects. This is probably due to the link between exercise and mood, confirming that in Parkinson’s disease, structured exercise programs are associated with improvements in mood-related outcomes, most consistently as reductions in depressive symptom severity and anxiety [34].

The strong spatiotemporal changes in sensorimotor rhythms observed across the gait cycle and the associated clinical improvement elicited by coupling music with gait training might depend on the precise modulation of dopamine release by internal and external timing mechanisms engaged by music, which enable fine-tuning of gait-cycle parameters to motor context and task demands in a manner reminiscent of levodopa and deep brain stimulation [4,26].

However, functional mobility outcomes varied across studies. Spina et al. [25] showed no immediate benefit and slight deterioration at 3-month follow-up, while Chawla et al. [28] demonstrated only short-term modulation of cadence during single-session treadmill conditions. In this scenario, a key finding is the transient nature of clinical gains, or the “washout effect.” Studies like Spina et al. [19] showed that benefits in gait speed and stride length might diminish after the end of intervention, suggesting that RAS could act as an external pacemaker requiring constant reinforcement. [14]. Consequently, music-based rehabilitation should be framed as a long-term strategy, incorporating “booster sessions” or home-based training to consolidate neuroplastic changes and prevent motor decay. This reflects the necessity of integrating the pRAS in a rehabilitation of PD, with programs including treadmill, resistance training, and structured balance and gait training or adapted physical activity, e.g., Nordic walking or Tai Chi [32,35,36,37].

Despite these encouraging results, heterogeneity in study design, personalization procedures, training duration, and outcome measures limits direct comparability across trials. This variability primarily stemmed from differences in intervention protocols, including duration (ranging from 4 to 24 weeks), session frequency, and the specific modalities of auditory stimulation employed, which ranged from basic metronomic cues to sophisticated music-contingent gait training [21,24,25,26,30]. Furthermore, baseline clinical characteristics varied significantly regarding disease severity (Hoehn & Yahr stages II–III) and cognitive status [22,29]. While such heterogeneity limited the definition of a single optimal protocol, it strengthened the external validity of the findings, suggesting that pRAS and music therapy remained effective across diverse clinical presentations. However, the data highlighted a critical need to standardize parameters to allow for precise meta-analytic conclusions. Most analyzed studies relied on small cohorts and lacked both long-term follow-up and systematic implementation [13,38]. Many studies involved small samples, lacked long-term follow-up, or did not systematically assess freezing of gait. Nevertheless, the methodological synthesis derived from the JBI analysis indicates that most studies demonstrated good to excellent methodological quality (scores 7–9/9); strengths included clear definition of intervention and outcomes, reliable measurement tools, and appropriate analytical approaches. The most frequent limitations related to incomplete follow-up (particularly in shorter interventions) and lack of control groups in selected experimental designs. These methodological considerations must be taken into account when interpreting effect sizes and determining the sustainability of benefits. Despite these challenges, adherence was consistently high (>85%), and no adverse events were reported, supporting the feasibility and safety of rhythmic-auditory interventions in both clinical and home-based contexts [39,40]. The progression from fixed-tempo cueing to personalized, contingent, and closed-loop systems illustrate a broader shift toward precision gait rehabilitation, where auditory stimuli are increasingly tailored to individual motor profiles, environmental conditions, and user preferences [41].

Taken together, the current evidence supports pRAS and MCGT as promising adjuncts to conventional gait rehabilitation in PD [42,43,44]. Future research should prioritize larger randomized controlled trials, harmonized personalization frameworks, and extended follow-up periods to determine optimal cueing parameters, evaluate durability of effects, and further elucidate the neurophysiological mechanisms underlying rhythmic entrainment. Integrating adaptive and closed-loop technologies into clinical practice may represent a critical step toward personalized, precision-based gait rehabilitation in Parkinson’s disease [45].

## 5. Conclusions

This systematic review showed that a personalized rehabilitation with pRAS and MCGT could be effective in improving gait performance and functional mobility in individuals with Parkinson’s disease. Integrating adaptive and closed-loop technologies into clinical practice may represent a critical step toward personalized, precision-based gait rehabilitation in Parkinson’s disease. From a clinical perspective, pRAS-based interventions appear particularly beneficial when integrated into structured gait rehabilitation programs, especially those combining treadmill training, balance exercises, and task-specific practice. Adaptive and closed-loop cueing systems may enhance motor entrainment, adherence, and patient engagement, supporting their feasibility in both clinical and home-based rehabilitation settings.

From a methodological perspective, current evidence supports the safety and short-term efficacy of pRAS; however, heterogeneity in intervention protocols and outcome measures limits the definition of standardized clinical guidelines.

Future research should prioritize larger randomized controlled trials with harmonized intervention parameters, standardized outcome measures, and longer follow-up periods to determine the durability of effects and to identify optimal cueing strategies for different clinical phenotypes.

## Figures and Tables

**Table 1:** Main characteristics of the randomized controlled trials included in the present systematic review.

Article	Nation	Study Groups	Intervention	Outcomes	Limitations	Main Findings
**de Bruin et al., 2010** [22]	Canada	22 PD patients, mild–moderate, HY II–III; Exp *n* = 11 gait training walking, Cnt *n* = 11 their regular daily activities	Exp: 13-week music therapy: 30 min, 3×/week with cadence-matched music; Cnt: usual activities	Gait velocity, stride time, stride length and cadence, dual-task errors, UPDRS III	Small sample; baseline heterogeneity; not active Cnt.; possible placebo; limited generalizability	MCGT, safe; improved gait parameters and UPDRS; greater effects in dual-task; no increase in falls
**Chaiwanichsiri et al., 2011** [23]	Thailand	30 male PD patients, HY II–III; 3 groups: A (*n* = 10) music + treadmill, B *(n* = 10) treadmill, C (*n* = 10) home-based gait training	4-week Group A: music + treadmill/home-based gait training; Group B treadmill/home-based gait training; Group C: home-based gait training. Every group followed with self practice for another 4 weeks.	Step/stride length, cadence, speed, 6MWD, TUG, ETUG, SLST	Only male patients; small sample; short follow-up; single blinded	Music-enhanced treadmill training led to greatest gains in gait parameters, sustained at 8 weeks
**Bukowska et al., 2016** [24]	Poland	55 PD patients (HY II–III); Exp *n* = 30 NMT, Cnt *n* = 25 as their usual daily activities	4-week NMT program, 45-min sessions, 4×/week; combined TIMP, PSE, RAS for ADL, pre-gait, gait, stability. Vs. usual activities.	Spatiotemporal gait parameters via BTS Smart; stability via CQStab posturography	Pilot study; cannot isolate individual effects of techniques; short-term only; no blinding;	Significant improvements in gait (step/stride length, velocity, cadence, swing phase); proprioception improved (eyes closed tests)
**Spina et al., 2016** [25]	Italy	25 PD patients with mild disability; randomized	24-week active MT, 1 session/week (90 min): music production, singing, dancing. Vs. usual activities.	TUG, MDS-UPDRS, PDQ-39, cognitive tests (FAB, Rey, fluency, TMT, Stroop)	Small sample, letters to the editor, pilot design, short-term follow-up, no active control	MT improved executive function, attention, memory, QoL; motor effects modest; benefits faded after discontinuation
**Burt et al., 2019** [21]	Canada	30 PD patients, mild–moderate PD; Exp *n* = 15 music played only when stride length met threshold; Cnt *n* = 15: with non-contingent music walking (constant music).	12-week Ambulosono * training; contingent: stride-length triggered music; control: 6 weeks noncontingent + 6 weeks contingent	UPDRS-III, GDS, BAI, 10 m walk single/dual, SMMSE, MoCA, HVLT-R, Stroop, TMT, ANT, DOT	Small sample; semi-randomized; short time (12 weeks); reporting bias, no follow-up	High adherence and safe; mood improved; no significant cognitive or motor changes; feasible intervention
**Calabrò et al., 2019** [26]	Italy	50 PD patients (HY II–III), randomized: Exp *n* = 25 RAS, Cnt *n* = 25 non-RAS	8-week rehab + 30 min/day treadmill; RAS: individualized rhythmic cues progressing to 120 bpm; control: treadmill without cues	Primary: FGA. Secondary: UPDRS-III, FES, BBS, 10MWT, TUG, GQI, EEG alpha/beta ERD/ERS e TRCoh	No follow-up; multimodal rehab may confound effects; EEG limited to treadmill; limited frequency bands; inpatient sample	RAS improved FGA, FES, UPDRS, TUG, GQI; stronger alpha/beta ERD-ERS and TRCoh; clinical gains correlated with EEG
**De Luca et al., 2020** [27]	Italy	40 PD patients (HY II–III, MMSE > 23); randomized: Exp *n* = 20 (music + treadmill), Cnt *n* = 20 (traditional overground gait training + standard physiotherapy).	8-week, 3×/week gait training; Experimental: Biodex Gait Trainer 3 with RAS (music + bell cue, up to 120 bpm); Control: overground gait training	PGWBI, Brief-COPE, FIM (total/cognitive/motor), TUG, 10 m Walk Test	Small sample, pilot design, no follow-up, inpatient sample, no instrumental neurophysiology	Music-assisted treadmill improved mood, coping, FIM, TUG, 10 mWT; greater gains vs control; feasible and effective
**Chawla et al., 2021** [28]	USA	21 PD patients (HY I–III). Within-subject crossover repeated-measures design.	Single-session treadmill: 7 conditions (no cue; metronome/music at 85%, 100%, 115% of cadence); participant-selected music matched by bpm	Cadence, step length, and cadence–cue accuracy, measured via Vicon motion capture and instrumented treadmill.	Music variability; handrail use; no cue–gait sync; only 1 min data/condition; small sample	Music has effects similar to the metronome; 85% cues reduced cadence and increased step length; 115% cues increased cadence and reduced step length; participants had difficulty matching slow targets.
**Fodor et al., 2021** [29]	Romania	32 PD patients (HY I–III); randomized into: Exp *n* = 16 (rehabilitation + music), Cnt *n* = 16 (rehabilitation only)	Exp: 2-week multimodal rehabilitation program, with classical music. After program instructed to continue listening to the same music 2.5 h/day for 2 more weeks. Cnt: rehabilitation program without music.	Activities of daily living (ADLs), emotional well-being, social support, communication, bodily discomfort.	Small sample size, Short duration (2-week intervention), Use of self-reported outcomes (PDQ-39), Music genre not individualized for all patients; Additional 2-week home listening may have influenced results.	Adding classical music to a multimodal rehabilitation program improved quality of life especially in ADLs, emotional well-being, communication, social support, and bodily discomfort.
**Porciuncula et al., 2025** [30]	USA	41 PD patients; randomized; Exp = 21 Amped-PD, Cnt = 20 Active-Control	6-week gait training; Amped-PD: MR-005 adaptive music-based RAS; Cnt: brisk walking without cues, both group followed with 2 weeks of self-practice	STV via 6-MWT, daily moderate-intensity walking minutes via SAM, steps/day, STV, gait speed/step length, SRHI, GRoC, UPDRS, Mini BESTest, 10-MWT, Self-Efficacy of Walking-D, ration4, stride velocity, stride length, Five-Times Sit-to-Stand Test, PDQ-39, Geriatric Depression Scale	Small sample, Short duration; baseline STV imbalance; single blinded	Amped-PD increased moderate-intensity walking and steps with large effect sizes; reduced STV; Cnt worsened; effects waned after device removal

Abbreviations: ADLs: Activities of daily living, bpm: beats per minute, Cnt: Control (referring to the control group), Exp: Experimental (referring to the experimental group), H&Y (or HY): Hoehn & Yahr (a scale used to classify the severity of PD), MCGT: Music-Contingent Gait Training, MMSE: Mini-Mental State Examination (a test used to screen for cognitive impairment), MT: music therapy, NMT: Neurologic Music Therapy, PD: Parkinson’s disease, PDQ-39: Parkinson’s Disease Questionnaire-39, PSE: Patterned Sensory Enhancement, QoL: quality of life, RAS: Rhythmic Auditory Stimulation, TIMP: Therapeutic Instrumental Music Performance. * Ambulosono is a gait-training method for Parkinson’s disease where a leg-mounted sensor controls music playback: the music plays only when the person takes sufficiently large steps and stops when steps become too short. This reward-based feedback motivates better stride length and helps improve walking quality, while being safe, enjoyable, and highly engaging.

**Table 2:** Certainty assessment of evidence for each outcome.

No. of Studies (Design)	RoB	Inconsistency	Indirectness	Imprecision	Other	Certainty
**Outcome: Spatiotemporal Gait Parameters (Speed, Stride Length)**						
10 (RCTs & Quasi-exp)	No serious concerns (0)	No serious concerns (0)	No serious concerns (0)	Serious (−1)	No serious concerns (0)	Moderate ⊕⊕⊕◯
**Outcome: Functional Mobility (Timed Up and Go)**						
4 (RCTs)	No serious concerns (0)	Serious (−1)	No serious concerns (0)	No serious concerns (0)	No serious concerns (0)	Moderate ⊕⊕⊕◯
**Outcome: Motor Severity (UPDRS-III)**						
5 (RCTs)	No serious concerns (0)	No serious concerns (0)	No serious concerns (0)	Serious (−1)	No serious concerns (0)	Moderate ⊕⊕⊕◯
**Outcome: Quality of Life (PDQ-39)**						
4 (RCTs & Pilot)	No serious concerns (0)	Serious (−1)	No serious concerns (0)	Very serious (−2)	No serious concerns (0)	Very low ⊕◯◯◯

**Certainty of Evidence (GRADE) Key:** ⊕⊕⊕⊕ **High:** We are very confident that the true effect lies close to that of the estimate of the effect. ⊕⊕⊕◯ **Moderate:** We are moderately confident in the effect estimate; the true effect is likely to be close to the estimate, but there is a possibility that it is substantially different. ⊕⊕◯◯ **Low:** Our confidence in the effect estimate is limited; the true effect may be substantially different from the estimate of the effect. ⊕◯◯◯ **Very low:** We have very little confidence in the effect estimate; the true effect is likely to be substantially different from the estimate of effect.

**Table 3:** Joanna Briggs Institute Critical Appraisal Checklist for quasi-experimental studies.

	Q1	Q2	Q3	Q4	Q5	Q6	Q7	Q8	Q9	Total Score
de Bruin et al., 2010 [22]	Y	Y	Y	Y	Y	Y	Y	Y	Y	9
Chaiwanichsiri et al., 2011 [23]	Y	Y	Y	Y	Y	Y	Y	Y	Y	9
Bukowska et al., 2016 [24]	Y	Y	Y	Y	Y	N	Y	Y	Y	8
Spina et al., 2016 [25]	Y	Y	Y	Y	Y	Y	Y	Y	Y	9
Burt et al., 2019 [21]	Y	Y	Y	Y	Y	N	Y	Y	Y	8
Calabrò et al., 2019 [26]	Y	Y	Y	Y	Y	Y	Y	Y	Y	9
De Luca et al., 2020 [27]	Y	Y	Y	Y	Y	N	Y	Y	Y	8
Chawla et al., 2021 [28]	Y	Y	N	N	Y	Y	Y	Y	Y	7
Fodor et al., 2021 [29]	Y	Y	Y	Y	Y	N	Y	Y	Y	8
Porciuncula et al., 2025 [30]	Y	Y	Y	Y	Y	Y	Y	Y	Y	9

Legend: Q1  =  Is it clear in the study what is the “cause” and what is the “effect” (i.e., there is no confusion about which variable comes first)?; Q2  =  Were the participants included in any comparisons similar?; Q3  =  Were the participants included in any comparisons receiving similar treatment/care, other than the exposure or intervention of interest?; Q4  =  Was there a control group?; Q5  =  Were there multiple measurements of the outcome both pre and post the intervention/exposure?; Q6  =  Was follow-up complete, and if not, were differences between groups in terms of their follow-up adequately described and analyzed?; Q7  =  Were the outcomes of participants included in any comparisons measured in the same way?; Q8  =  Were outcomes measured in a reliable way?; Q9  =  Was appropriate statistical analysis used?. N  =  no, Y  =  yes.

## Data Availability

The data presented in this study are available on request from the corresponding author.

## Supplementary Materials

The following supporting information can be downloaded at: https://www.mdpi.com/article/10.3390/neurolint18020026/s1, Supplementary File S1: PRISMA 2020 Checklist.

## Abbreviations

The following abbreviations are used in this manuscript:

10MWT (10-Meter Walk Test): A clinical test used to assess walking speed.

6MWD (6-Minute Walk Distance): A test to measure the maximum distance an individual can walk in 6 min to assess aerobic capacity and endurance.

ADLs (activities of daily living): Routine activities people do every day without assistance.

BBS (Berg Balance Scale): A widely used clinical test of a person’s static and dynamic balance abilities.

Cnt (Control): The group in an experiment or study that does not receive treatment by the researchers.

Exp (Experimental): The group in an experiment that receives the variable being tested.

FGA (Functional Gait Assessment): A clinical tool used to assess postural stability during various walking tasks.

FOG (Freezing of Gait): A clinical phenomenon where patients feel like their feet are glued to the ground, causing a brief inability to step forward.

H&Y/HY (Hoehn & Yahr): A commonly used system for describing how the symptoms of Parkinson’s disease progress.

JBI (Joanna Briggs Institute): An international research organization that provides a framework for evaluating the methodological quality of studies.

MCGT (Music-Contingent Gait Training): A rehabilitative approach where music playback is synchronized with or triggered by the patient’s walking movements.

MDS-UPDRS (Movement Disorder Society-Unified Parkinson’s Disease Rating Scale): The revised international standard for assessing Parkinson’s disease.

MMSE (Mini-Mental State Examination): A 30-point questionnaire that is used extensively in clinical and research settings to measure cognitive impairment.

MT (music therapy): The clinical and evidence-based use of music interventions to accomplish individualized goals.

NMT (Neurologic Music Therapy): The therapeutic application of music to cognitive, sensory, and motor dysfunctions due to neurologic diseases.

PD (Parkinson’s Disease): A progressive neurodegenerative disorder that primarily affects movement.

PDQ-39 (Parkinson’s Disease Questionnaire-39): A specific self-report questionnaire used to assess health-related quality of life in PD patients.

PICO (Participants, Intervention, Comparator, Outcome): A specialized framework used to form a clear and answerable clinical research question.

pRAS (Personalized Rhythmic Auditory Stimulation): An advanced form of auditory cueing tailored to the patient’s specific cadence and motor needs.

PRISMA (Preferred Reporting Items for Systematic reviews and Meta-Analyses): An evidence-based minimum set of items for reporting in systematic reviews and meta-analyses.

PSE (Patterned Sensory Enhancement): A technique using rhythmic, melodic, and harmonic patterns to provide temporal, spatial, and force cues for movement.

QoL (quality of life): The general well-being of individuals and societies, outlining negative and positive features of life.

RAS (Rhythmic Auditory Stimulation): A neurologic rehabilitation technique using rhythmic auditory cues to improve gait and movement.

RCT (Randomized Controlled Trial): A type of scientific experiment that aims to reduce certain sources of bias when testing the effectiveness of new treatments.

TIMP (Therapeutic Instrumental Music Performance): The use of musical instrument playing to facilitate engagement and exercise of functional motor patterns.

TUG (Timed Up and Go): A simple test used to assess a person’s mobility and requires both static and dynamic balance.

UPDRS (Unified Parkinson’s Disease Rating Scale): A rating tool used to gauge the severity and progression of Parkinson’s disease (Part III refers to the motor exam).
